# Characteristics of PM_2.5_ and Black Carbon Exposure Among Subway Workers

**DOI:** 10.3390/ijerph16162901

**Published:** 2019-08-13

**Authors:** Sangjun Choi, Ju-Hyun Park, So-Yeon Kim, Hyunseok Kwak, Dongwon Kim, Kyong-Hui Lee, Dong-Uk Park

**Affiliations:** 1 Department of Occupational Health, Daegu Catholic University, Gyeongsan 38430, Korea; 2 Department of Statistics, Dongguk University, Seoul 04620, Korea; 3 Department of Environmental Health, Korea National Open University, Seoul 03087, Korea; 4 Institute of Occupation and Environment, Korea Workers’ Compensation and Welfare Service, Incheon 21417, Korea; 5 Department of Environmental Health Sciences, Graduate School of Public Health, Seoul National University, Seoul 08826, Korea; 6 Force Health Protection & Preventive Medicine, MEDDAC-Korea, US Army, Seoul post 04386, Korea

**Keywords:** black carbon, PM_2.5_, subway, diesel engine motorcar

## Abstract

This study aimed to assess the characteristics of exposure to both PM_2.5_ and black carbon (BC) among subway workers. A total of 61 subway workers, including 26, 23, and 12 subway station managers, maintenance engineers, and train drivers, respectively, were investigated in 2018. Real-time measurements of airborne PM_2.5_ and BC were simultaneously conducted around the breathing zones of workers. Maintenance engineers had the highest average levels of exposure to both PM_2.5_ and BC (PM_2.5_, 76 µg/m^3^; BC, 9.3 µg/m^3^), followed by train drivers (63.2 µg/m^3^, 5.9 µg/m^3^) and subway station managers (39.7 µg/m^3^, 2.2 µg/m^3^). In terms of the relationship between mass concentrations of PM_2.5_ and BC, train drivers demonstrated the strongest correlation (R = 0.72), indicating that the proportion of BC contained in PM_2.5_ is relatively steady. The average proportion of BC in PM_2.5_ among maintenance engineers (13.0%) was higher than that among train drivers (9.4%) and subway station managers (6.4%). Univariate and mixed effect multiple analyses demonstrated the type of task and worksite to be significant factors affecting exposure levels in maintenance engineers and subway station managers. The use of diesel engine motorcars in tunnel maintenance was found to be a key contributor to PM_2.5_ and BC exposure levels among subway workers.

## 1. Introduction

In metropolitan areas, subways have become an indispensable form of transport as they reduce traffic congestion and improve air quality by reducing emissions from gasoline and diesel engines [1]. Recently, Lu et al. (2018) reported that adding new openings in the subway systems in Chinese cities between 2013 to 2017 reduced PM_2.5_ concentrations by an average of 18 µg/m^3^ and significantly improved air quality [2]. The Seoul Metropolitan subway is one of the largest metro traffic systems in the world. It comprises 22 lines, and serves Seoul, Incheon, and satellite cities in the Gyeonggi province. Most of the metropolitan subway stations in the Republic of Korea are situated deep underground. As metro traffic systems have expanded, there have been increasing concerns regarding underground air quality and the health risks of passengers [3,4].

Many studies have been conducted worldwide, including in the Republic of Korea, regarding the harmful substances and human health risks in the environment of the subway systems [5]. One of the major hazardous agents that both subway workers and passengers are exposed to is particulate matter (PM) of diverse sizes and chemical compositions. Smaller fractions of PM (PM_2.5_ and PM_1.0_) deserve particular attention as these particles may penetrate deep into the bronchiolar areas of the lung, causing various health hazards [6]. The PM generated in subway work environments include diesel engine exhaust (DEE) emissions, particles generated from friction during the movement of trains, and above ground PM infiltrating subway environments. Diesel engine vehicles are widely used for tunnel maintenance after completion of routine daily operations. The International Agency for Research on Cancer has reclassified DEE as “carcinogenic to humans (group 1)” based on sufficient evidence that exposure is associated with an increased risk of lung cancer [7].

Numerous studies have evaluated DEE exposures in various jobs using its primary surrogates (occasionally interchangeably), such as black carbon (BC), elemental carbon, total carbon, respirable particulate matter, and nitrogen dioxide. Studies have found that DEE exposures among underground miners and tunnel construction workers are markedly higher than those in aboveground truck/bus drivers, truck/bus garage mechanics, fire fighters, and heavy equipment operators [8,9,10,11,12,13]. However, few studies have assessed DEE exposure among subway workers; in particular, exposures to BC and PM_2.5_ have not been simultaneously evaluated in these workers.

The main sources of environmental BC are the incomplete combustion of fossil-based fuels and the burning of biomass on the Earth’s surface [14]. BC from vehicle exhausts originates from the combustion of diesel, gasoline, and other petroleum-based fuel materials that contain carbonaceous particles with attached polycyclic aromatic hydrocarbons (PAHs) [15]. Exhaust fumes from vehicles, including diesel engines operated both above ground and underground, could be the main source of BC in the subway environment. Exposure to BC has been closely linked with various adverse effects on health, including cancer development [16], lowered lung function [17], acute respiratory inflammation [18,19], wheezing [20], asthma exacerbation [21], decreased cognitive function [22], and attention problems [23].

According to the Korea Workers’ Compensation and Welfare Service, 10 cases of respiratory diseases (lung cancer = 8, idiopathic pulmonary fibrosis = 1, and chronic respiratory failure = 1) were reported from 2007 to 2012 as work-related diseases among subway workers [24]. However, studies on the air quality of subways have largely focused on its impact on the general population. Studies on workers who stay in the underground environment for longer periods than the general population are scarce. This study aimed to assess the daily exposure of subway workers to both PM_2.5_ and BC. It also aimed to evaluate the job- and subway-related environmental factors influencing PM_2.5_ and BC exposures and to evaluate the relationship between exposure to PM_2.5_ and BC.

## 2. Materials and Methods

### 2.1. Description of Subway Jobs

Subway workers in the Republic of Korea may be categorized into three types based on their jobs: maintenance engineers, subway station managers, and train drivers. Maintenance engineers may be further classified based on the various subway facilities that they manage, including equipment, machines, railways, and tunnels, among others. Exposure characteristics of hazardous agents among maintenance engineers may vary depending on the tasks they perform. The categories of maintenance work that this study focused on included the repair and maintenance of underground tunnels at night, after the completion of routine daily operations. These tasks are performed using diesel vehicles. Subway station managers mostly stay in offices located near ticket boxes and regularly patrol the entire length of the subway stations, including the platforms. They may be exposed to hazardous agents generated in subway stations that may vary depending on the location of the subway. Two shift work cycles operate between 5 a.m. to around midnight, from the beginning to the end of daily subway operations. Train drivers operate the trains according to irregular shift schedules, known as train diagrams. They work from small cabin rooms during the entire duration of their duty hours. Therefore, the characteristics of exposure to hazardous agents depends on the various facility characteristics of several stations along the subway route.

### 2.2. Exposure Assessment Strategy

A total of 61 subway workers, including 26 subway station managers, 23 maintenance engineers, and 12 train drivers working on the Seoul, Bundang, and Incheon city lines, were investigated between April and September 2018. The subway workers (n = 61) with shifts scheduled when the measurements were being conducted were all selected to participate in the study. The basic characteristics of the subway lines surveyed in this study are summarized in Table 1.

Real-time airborne PM_2.5_ and BC concentrations were both simultaneously monitored during working hours to capture differences in time-activity patterns, based on jobs and locations of on-duty workers. On the sampling day, workers were asked to carry two samplers for each PM_2.5_ and BC, equipped with a tube fitted near the breathing zone to estimate inhalational exposures. Simultaneously, a time-activity diary was completed by workers in a provided form. Information registered in the time-activity diary included the main tasks, the beginning and ending times for each job, and the locations they worked at or visited while on duty. The accuracy of information was verified by consulting the logged measurement levels.

### 2.3. PM_2.5_ Measurement

PM_2.5_ concentrations were measured using a SidePak personal aerosol monitor (Model AM510 or AM520, TSI Inc., Shoreview, MN, USA) fitted with a 2.5 μm impactor. The impactor was cleaned and greased prior to each use and was set to an airflow rate of 1.7 L/min. Prior to each measurement with the included high efficiency particulate air filter, the instrument was calibrated to zero. SidePak monitors are light-scattering laser photometers with a resolution of 1 µg/m^3^. The data obtained from the SidePak AM520 m were multiplied by a photometric calibration factor (PCF) of 0.38. The purpose of the PCF is to compensate when measuring for aerosols that have different photometric properties than those used during factory calibration. The manufacturer recommended that a PCF of 0.38 be used for the ambient aerosols present in the urban environment [25]. All SidePaks used in this study were calibrated by the manufacturer within the recommended yearly intervals. The recorded data were downloaded to personal computers and analyzed using TrakPro or TrakPro 5 software (TSI Inc.).

### 2.4. BC Measurement

The BC levels were monitored using an aethalometer (microAeth model AE51, Magee Scientific, Berkeley, CA, USA). This instrument measures the intensity of light (880 nm wavelength) transmitted through a T60 Teflon-coated glass fiber and reports BC concentrations in ng/m^3^. The detection limit of the aethalometer was 1 ng/m^3^. The manufacturer’s default specific attenuation coefficient of 16.6 m^2^/g was used. To enhance the sensitivity, the air sampling rate was set at 0.15 L/min. Real-time measurements were recorded every minute. Since the instrument was small and portable (280 g), it was possible to use it to directly monitor the daily personal exposure to BC while workers moved through different zones of activity with varying BC concentrations. The filter strips were replaced prior to each sampling to minimize the filter loading effect. To test the precision of monitoring, the results from four micro-aethalometers were intercompared.

### 2.5. Data Analysis

Real-time measurements of both PM_2.5_ and BC were simultaneously recorded for 1 min each during working hours. The data were downloaded soon after monitoring to minimize data handling errors or recall bias. The three data values of “0” in the BC measurements were changed to half of the detection limit of 1 ng/m^3^, as previously described [26]. A total of 14,085 measurements on one timescale were finally included in this study. The data pertaining to PM_2.5_ and BC were found to have a right-skewed distribution, indicating frequent high-level exposures. Therefore, the data were natural log-transformed for statistical analyses to better fit the normal distribution. The data were presented as descriptive statistics, including the arithmetic mean (AM), standard deviation (SD), geometric mean (GM), geometric standard deviation (GSD), minimum (Min) value, and maximum (Max) value. To identify work activities and potential environmental risk factors that contribute to exposure, all PM_2.5_ and BC records were classified according to the following several categories: (i) temporal factors: type of day, time of day, and rush hour; (ii) subway environmental factors: subway line, location of subway (underground or at ground level); and (iii) job: subway station manager, maintenance engineer, and train driver.

A linear mixed model was used to compare average natural logarithm-transformed PM_2.5_ and BC exposure levels according to categorized jobs, tasks, and environmental factors; correlations between repeated measurements at the same working or visited locations over time were modeled by a first-order autoregressive covariance structure with random effects. Sequential likelihood ratio tests (LRTs) were conducted to assess: (1) whether a linear mixed model was preferred for analyses over a linear regression model without random effects, and (2) the impact of potential risk factors on PM_2.5_ and BC exposures while accounting for subway features, such as line, on the basis of the test results of a random effect. For each model, residuals were thoroughly assessed to check the model’s assumptions, such as linearity and normality. The level of significance was set at 0.05 and all statistical analyses were performed using the R version 3.5.1. (R Foundation for Statistical Computing, Vienna, Austria) software package.

## 3. Results

### 3.1. PM_2.5_ and BC Exposure Characteristics

**Figure 1:**
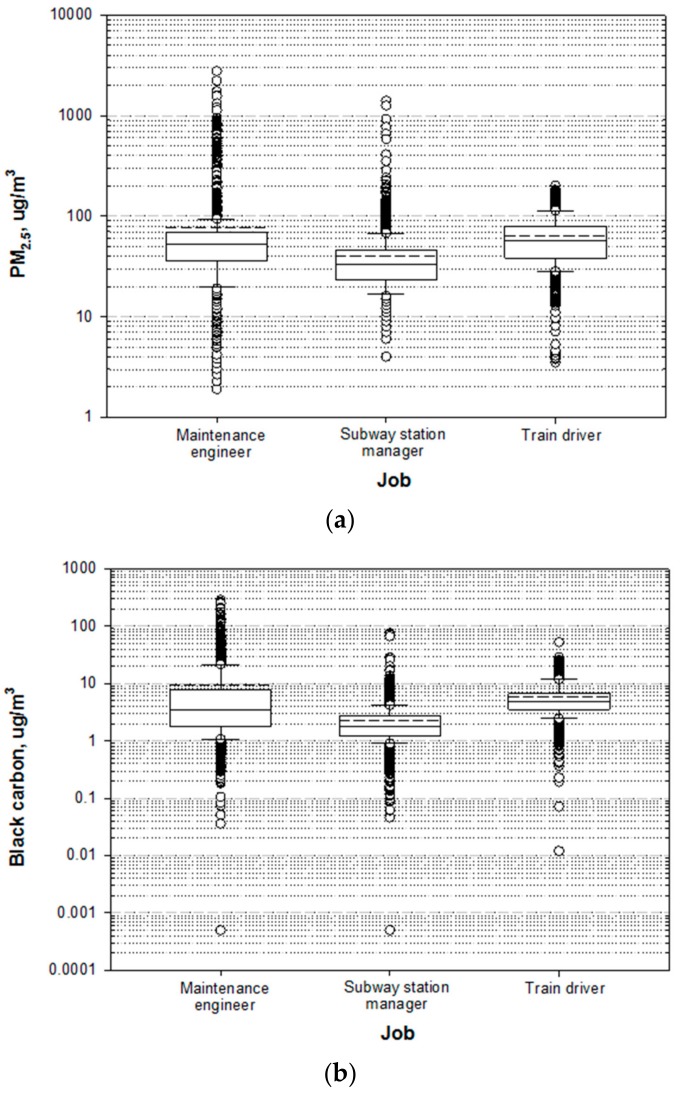
Distribution of (**a**) PM_2.5_ and (**b**) black carbon exposure levels according to job. Dashed lines show the arithmetic mean. * *p*-values were obtained using the likelihood ratio test. (**a**) PM_2.5_ (*p* < 0.001 *); (**b**) black carbon (*p* < 0.001 *).

The distribution of levels of exposure to PM_2.5_ and BC according to job are presented in Figure 1. The details of the association between exposure levels and subway environmental factors are summarized in Table 2.

The average PM_2.5_ and BC levels were 50.5 (range: 1.9–2774) µg/m^3^ and 4.3 (range: 0.0005–283.1) µg/m^3^, respectively. The exposure levels of both PM_2.5_ and BC were significantly different among the types of jobs (*p* < 0.0001) (Figure 1).

Maintenance engineers demonstrated the highest AM concentrations and the largest variations in both PM_2.5_ (AM = 76 µg/m^3^, SD = 139.2 µg/m^3^) and BC (AM = 9.3 µg/m^3^, SD = 19.3 µg/m^3^) (Table 2). Subway station managers showed the lowest average exposure levels to both PM_2.5_ (AM = 39.7 µg/m^3^, SD = 34.0 µg/m^3^) and BC (AM = 2.2 µg/m^3^, SD = 2.4 µg/m^3^). The GM of the concentrations of PM_2.5_ (54.9 µg/m^3^) and BC (4.9 µg/m^3^) was the highest among train drivers, with the lowest variation in PM_2.5_ (3.5–200.9 µg/m^3^).

In maintenance engineers and subway station managers, the type of task and the worksites where they spent their working hours were found to be the most significant factors affecting exposure to PM_2.5_ and BC. In subway station managers, the time variable was also found to contribute significantly to exposure levels of PM_2.5_ and BC (*p* < 0.001) (Table 2).

Among maintenance engineers and subway station managers, the linear mixed effects model also demonstrated task type to be the most significant factor associated with the exposure levels of PM_2.5_ and BC (*p* < 0.01) (Table 3). In maintenance engineers, a 10.3-fold higher GM of PM_2.5_ was seen with rail grinding than with gravel work, whereas in subway station managers, a 1.146-fold higher GM of PM_2.5_ was seen with patrolling than with monitoring; the observations for BC concentrations were similar. After adjusting for subway line in the linear mixed model, the time variable was not a significant factor for PM_2.5_ (*p* = 0.927) and BC (*p* = 0.287) exposure among train drivers.

### 3.2. Relationship between PM_2.5_ and BC Exposures by Job

**Figure 2:**
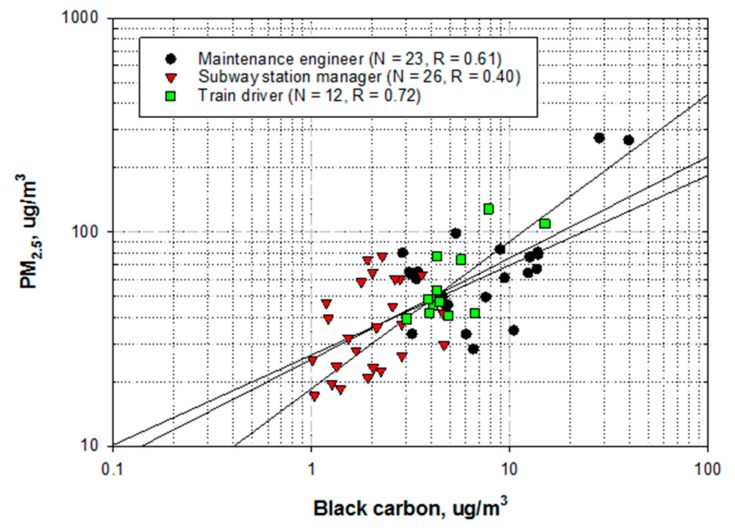
Correlation between the mass concentrations of PM_2.5_ and black carbon (BC) by job.

Figure 2 illustrates the correlation between the mass concentrations of PM_2.5_ and BC on the log scale by job. The correlation coefficients between jobs were calculated after conducting linear regression among 61 subjects. Train drivers demonstrated the strongest correlation (R = 0.72), followed by maintenance engineers (R = 0.61) and subway station managers (R = 0.4).

The proportion of BC contained in PM_2.5_ in the 61 subjects ranged from 2.5% to 30.2% (Table 4). The average proportion of BC in PM_2.5_ among maintenance engineers was significantly higher than that in the other jobs (maintenance engineers vs. subway station managers vs. train drivers: 13.0% vs. 6.4% vs. 9.4%).

**Figure 3:**
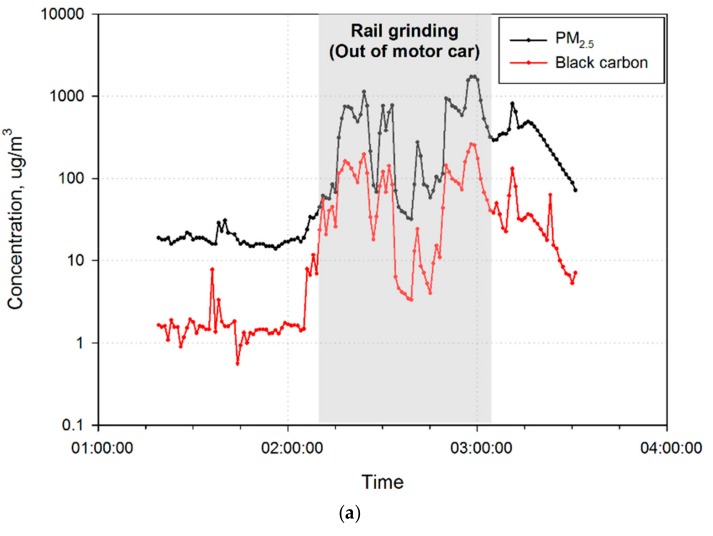

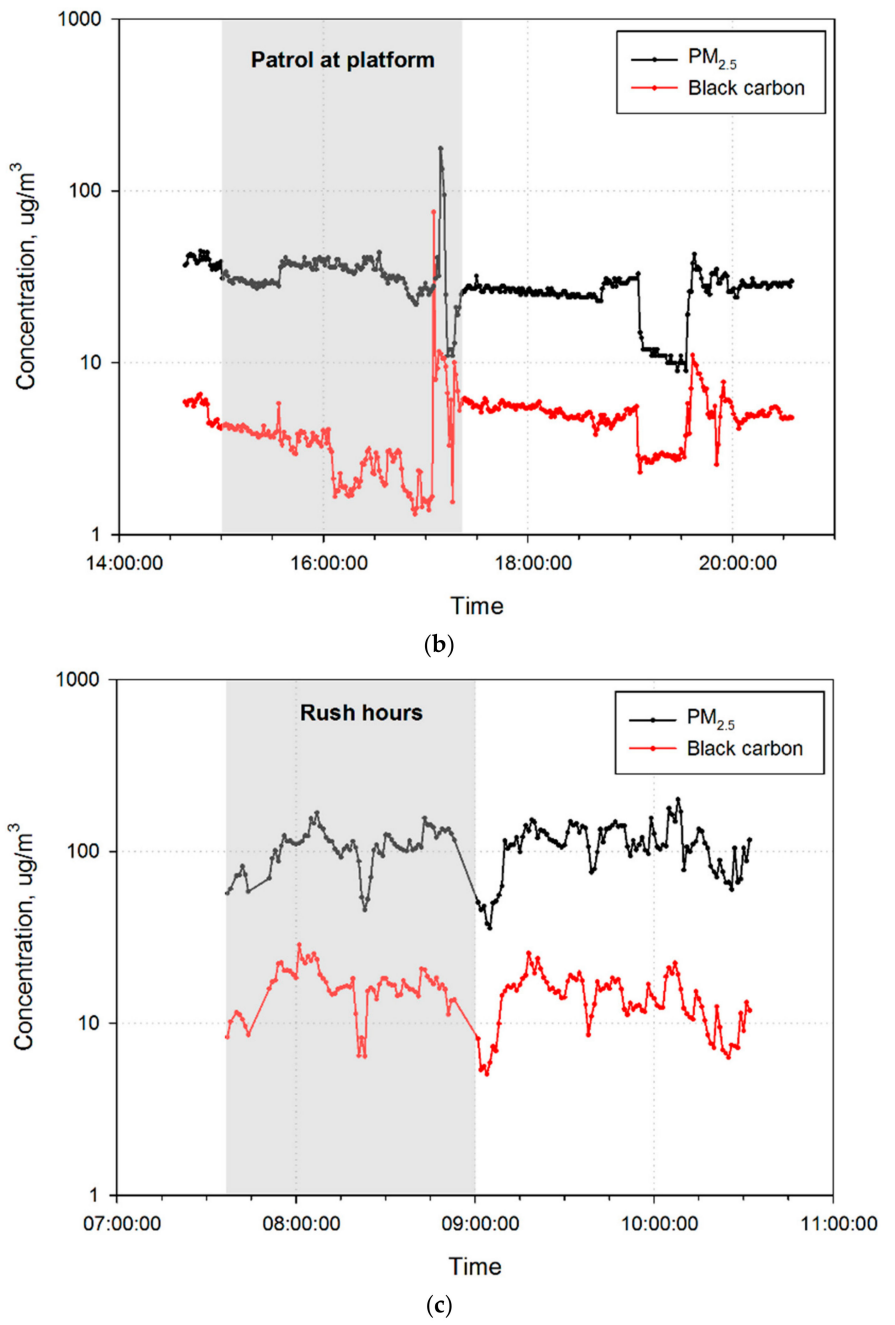
Real-time patterns of exposure to PM_2.5_ and BC according to job. (**a**) Maintenance engineers; (**b**) subway station managers; (**c**) train drivers.

Figure 3 displays the temporal exposure pattern of mass concentrations of PM_2.5_ and BC from data observed in representative cases, with the peak exposures by jobs. Several peaks of relatively high levels were generally observed in short periods of time during the entire work duration.

As seen in Figure 3a, peak exposures to PM_2.5_ and BC were higher in maintenance engineers than in the other workers. In particular, the PM_2.5_ and BC concentrations increased more than 100-fold during grinding of rails outside the diesel engine motorcar. As demonstrated in Figure 3b, the highest peak levels of PM_2.5_ (177.0 µg/m^3^) and BC (75.5 µg/m^3^) in subway station managers were observed during platform patrolling. As depicted in Figure 3c, train drivers showed relatively low diurnal variations in both PM_2.5_ and BC. Figure 3 demonstrates the correlation between the mass concentrations of PM_2.5_ and BC for each job, irrespective of tasks.

## 4. Discussion

This study assessed daily integrated levels of inhalational exposures to PM_2.5_ and BC among subway workers and explored the relationships between them according to jobs, tasks, locations, and time variables. Several factors were found to significantly influence the levels of exposure to PM_2.5_ and BC.

Type of job was found to be closely associated with levels of exposure to both PM_2.5_ and BC and with the relationship between PM_2.5_ and BC. Maintenance engineers demonstrated the highest levels of exposure to both PM_2.5_ and BC, followed by train drivers and subway station managers (Figure 1 and Table 2). The proportions of the mass concentrations of BC in PM_2.5_ indicated similar trends across the three jobs (Table 4). In terms of the relationship between the mass concentrations of PM_2.5_ and BC, train drivers showed the strongest correlation (R = 0.72), indicating that the proportion of BC contained in PM_2.5_ is relatively steady (Figure 2). The average proportion of BC in PM_2.5_ among maintenance engineers (13.0%) was higher than that among train drivers (9.4%) and subway station managers (6.4%). When the proportions of BC were monitored across 24 sites of EUROAIRNET in Europe; it was found to contribute to 5%–10% of PM_2.5_ and to lower proportions of PM with an aerodynamic diameter smaller than 10 μm (PM_10_) [27]. In particular, at some of the curbside sites, the proportion of BC in PM_2.5_ was 17%; this was similar to our results, which demonstrated a relative contribution of 16.5% among maintenance engineers who worked near diesel engine motor cars during rail grinding (Table 4).

In general, maintenance engineers use diesel engine motorcars while working inside tunnels. Since this maintenance work takes place at night when the electric power is switched off, the maintenance vehicle uses its own power; therefore, the diesel engines run throughout the duration of work. Our findings suggest that while working inside or close to diesel motorcars, maintenance engineers may be exposed to high levels of BC, exceeding 200 µg/m^3^ (Table 2). Interestingly, as seen in Figure 3a, the levels of exposure to BC during the rail grinding operation (54.7 µg/m^3^) were observed to be similar to the levels of exposure to PM_2.5_ (62.0 µg/m^3^) among all workers. These results demonstrate that maintenance engineers who maintain subway tunnels are generally exposed to high levels of DEE, with consequent high exposure to ultra-fine PM and BC. DEE contains respirable particles, of which 80%–95% are fine and measure <2.5 μm [28,29]. In this study, diesel particulate filters (DPFs) were not installed on all the diesel engine motorcars used by maintenance engineers. BC has been used as a surrogate indicator of DEE in the workplace [30,31]; PM_2.5_ may also be used to determine DEE exposures [32,33,34]. The use of diesel vehicles during tunnel maintenance was found to be a key contributor to the levels of exposure to BC and PM_2.5_ among maintenance engineers. This result is in agreement with a previous study conducted in New York City (NYC) [3]. The NYC study showed that BC levels increase rapidly when a diesel maintenance train is in operation nearby; therefore, diesel maintenance trains are potentially important contributors to elevated BC levels in subway stations.

A linear mixed model was employed as the main statistical analytic tool for this study; statistical tests for both random (initial test) and fixed effects (subsequent test) were conducted for LRTs [35]. For all the analyses presented in Table 2 and Table 3, linear mixed models fit the data better than linear regression models, implying that there were significant correlations among repeated PM_2.5_ or BC measurements. Readers should note that for a given analysis, both linear mixed and linear regression models had the same set of predictors, but differed only by a random effect. For the example of train drivers’ exposure to PM_2.5_ in Table 3, variables Time2 and line were included as fixed effects in both models and, based on the LRT result that a linear mixed model had a better fit, the effect of Time2 on log-transformed PM_2.5_ was evaluated with another LRT in the presence of line as a covariate. Although data were not shown, no remarkable pattern was observed in the residual assessment of linear mixed models, indicating that model assumptions seemed to be satisfied.

Comparisons of PM_2.5_ and BC exposure levels according to job, tasks, worksites, and time provide accurate estimates of daily exposure levels. This study assessed the exposure of individual subway workers, so we did not evaluate air quality by sampling fixed areas at the platform or station hall. Therefore, workers’ job characteristics were more important factors in understanding their exposure levels than the features of the subway line, such as the year it opened or the number of stations. Both univariate and linear mixed effect multiple analyses revealed tasks and worksites to be significant factors affecting exposure levels among maintenance engineers and subway station managers (Table 2 and Table 3). Subway station managers showed the highest exposure levels during platform patrolling near tunnels, in which diesel engine motorcars were the primary sources of PM_2.5_ and BC. In train drivers, the PM_2.5_ and BC exposure pattern did not follow the general trend; the exposure to hazardous agents, including PM, did not increase with an increase in traffic volumes near the subway. Above ground, traffic volume directly affects the exposure level of commuters. For instance, Jereb et al. showed that traffic air pollution significantly influences cyclists riding near main roads [36]. Many studies have found that underground air pollutant concentrations in subways are higher than aboveground levels [3,37,38]. Therefore, the pollutant concentration in underground air is affected not only by the amount of traffic above ground but also by ventilation in the subway system [39,40]. Among train drivers, the average levels of exposure to PM_2.5_ and BC were comparatively lower during the rush hours than during other times (Table 2). However, as shown in Figure 3c, this difference was not significant. Similar results can be seen in the Shanghai subway study [38]. In the Shanghai study, due to the greater train frequency and passenger volume, the average PM_2.5_ levels at underground platforms during the rush hour period were significantly higher than those during the non-rush hour period. However, on clear days, the average PM_2.5_ levels in station halls during the rush hour period were significantly lower than those during the non-rush hour period. The researchers of the Shanghai study suggested that one possible explanation for this phenomenon could be increased air exchange caused by the stronger piston wind between the station hall and the outside environment at entrances/exits during the rush hour period. We think this could also be an explanation for the train drivers’ exposure pattern observed in this study. The exposure level of train drivers may have been especially sensitive to the ventilation inside the tunnel because train drivers stayed in subway cabins for the entire sampling period. 

No limits have been set for occupational exposures to PM_2.5_ and BC. The Korean Ministry of Environment set the standard average value for the atmospheric levels of PM_2.5_ over 24 h and one year to be 35 and 15 µg/m^3^, respectively [41]. In addition, for the first time, the Korean Indoor Air Quality standard for PM_2.5_ in subway stations is planned to be set at a daily average of 50 µg/m^3^ from 2019 [42]. We speculate that the use of diesel engine vehicles for tunnel maintenance significantly contributes to the PM_2.5_ and BC exposures among workers and passengers in the subway system. Vilcassim et al. suggested that diesel maintenance trains are a potentially important contributor to elevated BC levels in subway stations and that PM traps should be applied to diesel engines as one potential mitigation measure [3]. Our findings also indicate that installation of DPFs on diesel engine maintenance vehicles is warranted.

This study has several limitations. A major drawback lies in the uncertainty of whether the BC exposure levels among subway workers in this cohort are representative of all subway systems. These findings may not be generalizable to other subway environments with different workplace characteristics and physical environments in and around the subway. Since the 61 subway workers surveyed were not randomly selected, the PM_2.5_ and BC concentrations measured cannot be generalized as representative values for each job. The jobs and work patterns among subway workers may not be representative of those who work in environments with variable characteristics, such as depths, levels of ventilation, and transport modes surrounding the subway. The composition and generation of DEE varies depending on the age of the diesel engine, type of engine, fuel characteristics, driving cycle, and filtration of the exhaust. In addition, our monitoring period (from late spring to summer) did not encompass all the seasons of the year; this may also reduce the generalizability of our findings. Our data in the summer included personal PM_2.5_ and BC levels measured between April and September; this may have underestimated the daily BC exposure levels in the colder months, when reinforced heating and reduced ventilation are likely to increase exposure. Fromme et al reported that the concentrations of benzo-a-pyrene and elemental carbon monitored in the winter are three to four times higher than those in the summer, corresponding to the changes in ambient air concentrations in cars and subway transportation systems [43].

This study has certain strengths. It identified job type and use of diesel engine vehicles for tunnel maintenance to be potential risk factors for PM_2.5_ and BC exposures among subway workers. To our knowledge, this study is the first to simultaneously assess the levels of exposure to both PM_2.5_ and BC among subway workers by using synchronous real-time personal monitoring.

## 5. Conclusions

In conclusion, both the PM_2.5_ and BC exposure levels for subway workers were significantly different according to the type of job, the tasks conducted, and the worksite. Maintenance engineers had the highest exposure to both PM_2.5_ and BC, followed by train drivers, then subway station managers. In terms of the tasks and worksite, maintenance engineers and subway station managers showed the highest exposure levels during rail grinding in tunnels with diesel engine motorcars and patrolling platforms close to tunnels, respectively. This study also shows that the use of diesel engine motorcars during tunnel maintenance is a key contributor to PM_2.5_ and BC exposures among subway workers. Therefore, proactive measures, including installing DPFs on diesel engine maintenance vehicles in tunnels, are urgently needed to reduce subway workers’ exposure to both PM_2.5_ and BC. Further studies on other subway systems are needed to validate our findings.

## Figures and Tables

**Table 1:** Basic characteristics of subway lines surveyed in this study.

Region	Line	Operating Company	Opening Year	Number of Stations
Seoul	1	Seoul Metro	1974	10
	1 (Gyeongbu line)	KORAIL	1974	37
	1 (Gyeongin line)	KORAIL	1974	21
	1 (Gyeongwon line)	KORAIL	1974	25
	1 (Janghang line)	KORAIL	2008	7
	2	Seoul Metro	1980	44
	2 (Seongsu branch line)	Seoul Metro	1980	5
	2 (Sinjeong branch line)	Seoul Metro	1992	5
	3	Seoul Metro	1985	34
	3 (Ilsan line)	KORAIL	1996	11
	4	Seoul Metro	1985	26
	4 (Gwacheon line)	KORAIL	1993	10
	4 (Ansan line)	KORAIL	1988	14
	5	Seoul Metro	1995	44
	5 (Macheon branch line)	Seoul Metro	1996	8
	6	Seoul Metro	2000	38
	7	Seoul Metro	1996	51
	9	Metro 9	2009	30
Seoul, Gyeonggi	Bundang	KORAIL	1994	36
Incheon	1	Incheon Transit Corporation	1999	29

**Table 2:** Summary of exposure levels to PM_2.5_ and black carbon according to job and task.

Job		PM_2.5_, µg/m^3^									BC, µg/m^3^						
		N. sub	N. meas	AM	SD	GM	GSD	Min	Max	*p*-value *	AM	SD	GM	GSD	Min	Max	*p*-value
Maintenance engineers		23	3173	76.0	139.2	49.2	2.2	1.9	2774.0		9.3	19.3	4.0	3.3	0.0005	283.1	
Line	Incheon 1	5	713	40.5	17.7	37.4	1.5	19.0	202.0	0.106	5.1	5.4	3.2	3.0	0.0005	49.6	0.009
	Seoul 2	6	1281	62.9	124.8	47.5	1.9	5.0	2774.0		7.0	10.2	3.7	2.9	0.036	84.0	
	Seoul 3	5	420	75.0	23.8	71.5	1.4	36.0	166.0		12.5	15.9	6.3	3.4	0.21	86.0	
	Seoul 4	3	421	184.6	288.3	47.5	5.7	1.9	1738.0		25.3	42.8	7.0	5.4	0.34	283.1	
	Seoul 9	4	338	66.9	10.8	66.0	1.2	38.0	90.0		3.2	2.0	2.8	1.7	0.64	15.4	
Worksites	Inside motorcar	15	1407	77.2	122.4	45.8	2.6	1.9	866.4	<0.001	9.1	18.3	3.9	3.4	0.0005	283.1	<0.001
	Outside motorcar	18	1766	75.1	151.3	52.1	2.0	5.0	2774.0		9.4	20.0	4.2	3.2	0.036	263.3	
Task	Gravel work	2	438	44.8	26.2	37.0	1.9	5.0	163.0	<0.001	8.0	8.4	5.0	2.7	0.19	52.7	<0.001
	Moving	11	778	79.7	84.3	57.9	2.3	1.9	832.0		10.2	19.2	4.5	3.3	0.21	283.1	
	Preparation	19	1724	71.9	143.0	46.3	2.1	2.7	2774.0		7.5	14.0	3.4	3.2	0.0005	248.9	
	Rail grinding	1	55	469.2	457.9	253.3	3.5	32.0	1738.0		77.5	68.2	42.5	3.7	3.34	263.3	
	Watering	2	178	55.1	14.2	53.3	1.3	27.0	104.0		5.4	5.8	3.5	2.5	0.51	28.3	
Subway station manager		26	9330	39.7	34.0	33.7	1.7	4.0	1402.0		2.2	2.4	1.8	1.9	0.0005	75.5	
Line	Bundang	2	644	31.8	16.7	29.5	1.5	8.0	353.0	0.006	2.9	3.9	2.3	2.2	0.046	70.6	0.182
	Incheon 1	2	622	69.4	76.9	59.1	1.6	29.0	1402.0		2.0	4.4	1.6	1.6	0.14	73.2	
	Seoul 3	6	2177	49.8	20.8	46.2	1.5	9.0	225.0		2.9	2.4	2.4	1.9	0.0005	75.5	
	Seoul 4	4	1267	42.1	27.6	35.8	1.8	9.0	410.0		2.7	1.9	2.2	2.0	0.23	28.4	
	Seoul 5	3	1178	20.6	5.9	19.8	1.3	4.0	60.0		1.3	0.8	1.2	1.6	0.0005	17.2	
	Seoul 6	3	1343	34.4	12.4	32.4	1.4	15.0	76.0		1.7	0.7	1.5	1.6	0.062	4.7	
	Seoul 7	2	644	50.0	30.0	41.0	1.9	14.0	120.0		2.2	0.9	2.0	1.5	0.20	10.3	
	Seoul 9	4	1455	28.7	42.9	24.1	1.6	9.0	1256.0		1.9	2.5	1.5	1.9	0.11	66.4	
Location	Ground	5	151	49.0	115.7	29.1	2.2	10.0	1256.0	0.025	1.8	1.3	1.5	1.8	0.53	11.3	0.327
	Underground	26	9179	39.5	30.9	33.8	1.7	4.0	1402.0		2.3	2.4	1.8	1.9	0.0005	75.5	
Worksite	Office	26	5495	36.1	21.2	31.9	1.6	7.0	410.0	<0.001	2.2	2.0	1.8	1.9	0.0005	70.6	<0.001
	Outdoors	5	151	49.0	115.7	29.1	2.2	10.0	1256.0		1.8	1.3	1.5	1.8	0.53	11.3	
	Passageway	26	2812	39.8	26.6	33.7	1.8	4.0	583.0		2.1	2.7	1.7	1.8	0.046	73.2	
	Platform	20	872	59.8	66.8	48.0	1.9	9.0	1402.0		3.2	3.2	2.6	1.9	0.19	75.5	
Time	Rush hour **	26	3133	35.4	18.4	31.7	1.6	6.0	353.0	<0.001	2.1	2.3	1.8	1.8	0.14	73.2	<0.001
	Others	26	6197	41.8	39.4	34.7	1.8	4.0	1402.0		2.3	2.4	1.8	2.0	0.0005	75.5	
Task	Monitoring	26	5412	35.8	20.9	31.7	1.6	7.0	410.0	<0.001	2.2	2.0	1.8	1.9	0.0005	70.6	<0.001
	Patrolling	26	3687	45.3	46.6	36.9	1.9	4.0	1402.0		2.4	2.9	1.9	1.9	0.046	75.5	
	Rest	10	231	40.1	29.6	32.6	1.9	10.0	207.0		1.8	1.2	1.5	1.8	0.086	10.0	
Train driver		12	1582	63.2	33.0	54.9	1.7	3.5	200.9		5.9	4.2	4.9	1.9	0.012	52.5	
Line	Bundang	1	96	39.3	9.2	38.1	1.3	20.5	54.8	0.181	3.0	0.9	2.8	1.9	0.012	5.4	0.003
	Incheon 1	2	218	44.3	11.9	42.6	1.3	20.0	68.2		4.6	1.6	4.2	1.7	0.071	8.4	
	Seoul 1	1	171	77.3	16.3	75.7	1.2	48.7	120.9		4.3	1.4	4.1	1.4	1.79	8.6	
	Seoul 2	1	179	42.0	24.1	34.6	2.0	3.5	99.2		6.6	3.5	5.5	2.0	0.37	13.9	
	Seoul 3	1	162	108.9	30.1	104.1	1.4	35.8	200.9		15.0	4.7	14.2	1.4	5.06	28.6	
	Seoul 4	2	267	75.7	46.4	60.6	2.0	13.5	182.7		5.5	4.1	4.4	2.0	0.19	52.5	
	Seoul 5	1	185	45.0	17.3	42.0	1.5	19.3	93.5		4.1	1.4	3.9	1.4	1.89	7.1	
	Seoul 7	1	171	74.3	9.8	73.7	1.1	55.7	98.1		5.7	1.6	5.4	1.4	1.79	10.9	
	Seoul 9	2	133	51.5	11.4	50.4	1.2	29.0	118.9		4.1	1.0	4.0	1.3	1.26	7.3	
Time 1	Rush hour	12	824	57.8	28.4	50.6	1.7	3.5	168.8	<0.001	5.6	4.0	4.7	1.9	0.071	28.6	0.587
	Others	10	758	69.0	36.5	60.0	1.7	13.5	200.9		6.3	4.4	5.1	2.0	0.012	52.5	
Time 2	Forenoon	6	862	68.9	31.1	61.0	1.7	3.5	200.9	0.446	7.1	4.8	5.9	1.8	0.37	28.6	0.082
	Afternoon	6	720	56.3	33.9	48.5	1.7	13.5	182.7		4.5	2.9	3.9	1.9	0.012	52.5	
Total		61	14085	50.5	74.1	38.7	1.9	1.9	2774.0		4.3	9.9	2.4	2.5	0.0005	283.1	

Abbreviations: N. sub: number of subjects, N. meas: number of measurements, AM: arithmetic mean, SD: standard deviation, GM: geometric mean, GSD: geometric standard deviation, Min: minimum, Max: maximum. * *p*-values were obtained using the likelihood ratio test considering correlations between measurements on the same subject. ** Rush hour: 07–09 a.m. to 06–08 p.m.

**Table 3:** Multiplicative effects of factors on the GM of PM_2.5_ and BC exposure levels using linear mixed model analysis ^†^.

Job	Variable	Level	PM_2.5_, µg/m^3^			BC, µg/m^3^		
			Estimate	SE	*p*-Value ****	Estimate	SE	*p*-Value
Maintenance engineers	Task *	Moving	1.124	1.454	<0.001	0.497	1.457	0.007
		Preparation	1.425	1.448		0.633	1.440	
		Rail grinding	10.328	1.752		2.964	2.276	
		Watering	1.411	1.661		0.751	1.655	
Subway station managers	Task **	Patrolling	1.146	1.007	<0.001	1.165	1.009	<0.001
		Rest	1.073	1.024		0.996	1.031	
Train drivers	Time 2 ***	Afternoon	1.023	1.843	0.927	0.852	1.426	0.287

^†^ For each job-specific analysis, a linear mixed model was found to be a better fit than a linear regression model (*p* < 0.001). * The reference level was “gravel work”; line and worksite were adjusted in the linear mixed model. ** The reference level was “monitoring”; line, worksite, and time were adjusted in the linear mixed model. *** The reference level was “forenoon”; line was adjusted in the linear mixed model. **** The *p*-value of the likelihood ratio test has been reported.

**Table 4:** Mass concentrations of BC in PM_2.5_ according to job and tasks.

Job *	Task	Black Carbon in PM_2.5_, %			
		N. sub	AM	Min	Max
Maintenance engineers	Subtotal	23	13.0	3.6	30.2
	Gravel work	2	19.3	15.4	23.1
	Moving	11	13.6	3.6	26.3
	Preparation	19	11.5	3.3	36.9
	Rail grinding	1	16.5	16.5	16.5
	Watering	2	10.4	5.6	15.3
Subway station managers	Subtotal	26	6.4	2.5	15.7
	Monitoring	26	6.4	1.9	18.2
	Patrolling	26	6.4	2.1	13.4
	Rest	10	4.8	1.9	8.8
Train drivers	Subtotal	12	9.4	5.6	15.8
	Driving	12	9.4	5.6	15.8
Total		61	9.5	2.5	30.2

Abbreviations: N. sub: number of subjects, AM: arithmetic mean, Min: minimum, Max: maximum. * *p* < 0.001 on analysis of variance.
